# Sequential Accumulation of ‘Driver’ Pathway Mutations Induces the Upregulation of Hydrogen-Sulfide-Producing Enzymes in Human Colonic Epithelial Cell Organoids

**DOI:** 10.3390/antiox11091823

**Published:** 2022-09-15

**Authors:** Kelly Ascenção, Nahzli Dilek, Karim Zuhra, Katalin Módis, Toshiro Sato, Csaba Szabo

**Affiliations:** 1 Chair of Pharmacology, Faculty of Science and Medicine, University of Fribourg, 1700 Fribourg, Switzerland; 2 Department of Surgery, University of Texas Medical Branch, Galveston, TX 77555, USA; 3 Department of Organoid Medicine, Keio University School of Medicine, Tokyo 160-8582, Japan

**Keywords:** Vogelstein sequence, colon cancer, hydrogen sulfide, cystathionine beta-synthase, 3-mercaptopyruvate sulfurtransferase

## Abstract

Recently, a CRISPR-Cas9 genome-editing system was developed with introduced sequential ‘driver’ mutations in the WNT, MAPK, TGF-β, TP53 and PI3K pathways into organoids derived from normal human intestinal epithelial cells. Prior studies have demonstrated that isogenic organoids harboring mutations in the tumor suppressor genes APC, SMAD4 and TP53, as well as the oncogene KRAS, assumed more proliferative and invasive properties in vitro and in vivo. A separate body of studies implicates the role of various hydrogen sulfide (H_2_S)-producing enzymes in the pathogenesis of colon cancer. The current study was designed to determine if the sequential mutations in the above pathway affect the expression of various H_2_S producing enzymes. Western blotting was used to detect the expression of the H_2_S-producing enzymes cystathionine β-synthase (CBS), cystathionine γ-lyase (CSE) and 3-mercaptopyruvate sulfurtransferase (3-MST), as well as several key enzymes involved in H_2_S degradation such as thiosulfate sulfurtransferase/rhodanese (TST), ethylmalonic encephalopathy 1 protein/persulfide dioxygenase (ETHE1) and sulfide-quinone oxidoreductase (SQR). H_2_S levels were detected by live-cell imaging using a fluorescent H_2_S probe. Bioenergetic parameters were assessed by Extracellular Flux Analysis; markers of epithelial-mesenchymal transition (EMT) were assessed by Western blotting. The results show that the consecutive mutations produced gradual upregulations in CBS expression—in particular in its truncated (45 kDa) form—as well as in CSE and 3-MST expression. In more advanced organoids, when the upregulation of H_2_S-producing enzymes coincided with the downregulation of the H_2_S-degrading enzyme SQR, increased H_2_S generation was also detected. This effect coincided with the upregulation of cellular bioenergetics (mitochondrial respiration and/or glycolysis) and an upregulation of the Wnt/β-catenin pathway, a key effector of EMT. Thus sequential mutations in colon epithelial cells according to the Vogelstein sequence are associated with a gradual upregulation of multiple H_2_S generating pathways, which, in turn, translates into functional changes in cellular bioenergetics and dedifferentiation, producing more aggressive and more invasive colon cancer phenotypes.

## 1. Introduction

In 2013, the concept that cancer cells overexpress the transsulfuration enzyme cystathionine β-synthase (CBS) and use its enzymatic product, hydrogen sulfide (H_2_S), was formulated, first in colon cancer [1] and subsequently in ovarian cancer [2]. In the following decade, the field of H_2_S and cancer has expanded significantly. The overexpression of H_2_S-producing enzymes (CBS, but also 3-mercaptopyruvate sulfurtransferase, 3-MST and cystathionine γ-lyase, CSE) has been detected in many different types of cancer; known cancer-supporting roles of H_2_S include the stimulation of cellular bioenergetics, the induction of proliferative and cytoprotective pathways, the stimulation of tumor angiogenesis, the promotion of epithelial to mesenchymal transition (EMT), protection against immune-cell-mediated elimination, maintenance of cancer cell stemness and others (reviewed in [3]).

Colon carcinogenesis, according to the universally accepted concept, is a result of consecutive mutations that accumulate in the colonic epithelial cells and transform the normal epithelial cells to epithelial polyps (through a mutation in the APC gene), and subsequent mutations (the most significant ones being designated as ‘driver’ mutations) transform the cells into carcinoma in situ, followed by local and, eventually, invasive cancer [4,5,6,7].

The relationship of these mutations to the changes in the expression of various enzymes involved in the regulation of H_2_S biogenesis and degradation in cancer cells has not yet been directly investigated. Here we have utilized a series of isogenic human organoids with the introduction of sequential accumulating driver mutations [8] to address this question.

## 2. Materials and Methods

### 2.1. Cell Culture

The organoids containing various driver mutations described in Table 1 (NL, A, AT, AKST, Ade^CIN^ TS and Ade^CIN^ TSK) were generated as previously described [8] using Corning^®^ Matrigel^®^ Basement Membrane Matrix (Corning Inc., Corning, NY, USA) and IntestiCult™ Organoid Growth Medium (Human) (StemCELL Technologies, Vancouver, BC, Canada) supplemented with 100 ng/mL of human IGF-I (Peprotech, Rocky Hill, NJ, USA) and 100 ng/mL of human FGF-basic (Peprotech).

### 2.2. Reagents and Antibodies

The CBS/CSE inhibitor AOAA (O-(carboxymethyl)hydroxylamine hemihydrochloride) [9] was purchased from Sigma-Aldrich (Burlington, MA, United States), the 3-MST inhibitor HMPSNE (2-[(4-hydroxy-6-methylpyrimidin-2-yl)sulfanyl]-1-(naphthalen-1-yl)ethan-1-one) [10] was purchased from MolPort (Riga, Latvia). The fluorescent H_2_S probe 7-azido-4-methylcoumarin (AzMC) was obtained from Sigma-Aldrich (Burlington, MA, USA).

Rabbit monoclonal anti-CBS (D8F2P), anti-ATP citrate lyase (ACLY) (D1 × 6P), anti-β-catenin (D10A8), anti-mouse IgG HRP-linked antibody (#7076) and rabbit polyclonal anti-phospho-ACLY (Ser455) were purchased from Cell Signaling Technology (Danvers, MA, USA). Mouse monoclonal anti-β-actin (AC-15) and rabbit polyclonal anti-SQR (HPA017079) were obtained from Sigma-Aldrich (Saint Louis, MO, USA). Anti-rabbit IgG (H+L) cross-adsorbed secondary antibody-HRP (#31458) was purchased from Invitrogen (Thermo Fisher Scientific, Waltham, MA, USA). Rabbit polyclonal anti-3-MST (ab224043), anti-CSE (ab151769), anti-TST (ab231248) and rabbit monoclonal anti-ETHE1 (ab174302) were purchased from Abcam (Cambridge, UK).

### 2.3. Western Blotting

The organoids were dissociated from Corning^®^ Matrigel^®^ Basement Membrane Matrix (Corning) using TrypLE™ Express Enzyme (Gibco, Thermo Fisher Scientific) and lysed with RIPA lysis buffer (Thermo Fisher Scientific) supplemented with Halt™ Protease and Phosphatase Inhibitor Cocktail (Thermo Fisher Scientific) and sonicated for 15 s in an ultrasonic water bath (XUBA3, Grant, Cambridge, UK). Measurement of protein concentrations, gel electrophoresis, proteins transfer, antibody incubations, development and quantification of the blots were performed as described [11]. Intensity values of related bands were normalized to β-actin housekeeping protein values. Representative blots of at least four independent experiments are shown.

### 2.4. Quantification of H_2_S Production

The organoids NL, A, AT, Ade^CIN^ TS and Ade^CIN^ TSK were seeded in Corning^®^ Matrigel^®^ Basement Membrane Matrix (Corning) in a 48-well plate. After 7 days of culture supernatant was replaced by 100 μM of AzMC diluted in HBSS buffer (Gibco, Thermo Fisher Scientific) and the cells were incubated 1 h at 37 °C and 5% CO_2_. The excitation (365 nm) and emission (450 nm) wavelength of AzMC were read in an Infinite 200 Pro reader (Tecan, Männedorf, Switzerland) as described [12]. Fluorescence values were normalized with total proteins values. The AzMC assays were performed at least 4 times in duplicates.

### 2.5. Proliferation Assays

The proliferation of the organoids NL, A, AT, Ade^CIN^ TS and Ade^CIN^ TSK after 7 days of culture was measured using the Cell Proliferation Elisa BrDU (colorimetric) kit purchased from Sigma-Aldrich (Saint Louis, MO, USA) as described [13]. Values were normalized to total cellular protein concentration. The proliferation rate of Ade^CIN^ TS and Ade^CIN^ TSK incubated with various concentrations of HMPSNE or AOAA was determined. Proliferation assays were performed at least 4 times in duplicates.

### 2.6. Cellular Bioenergetic Measurements

The organoids NL, A, AT, Ade^CIN^ TS and Ade^CIN^ TSK cellular bioenergetics were measured by extracellular flux analysis method using a Seahorse XFe-24 flux analyzer (Agilent Technologies, Santa Clara, CA, USA) as previously described [13,14]. The cells were seeded in Corning^®^ Matrigel^®^ Basement Membrane Matrix (Corning) in a Seahorse XFe-24 Cell Culture Microplates and incubated at 37 °C and 5% CO_2_. For the mitochondrial respiration assay, after 7 days of culture cells were washed twice with Seahorse XF DMEM (Agilent Technologies) supplemented with 20 µM of L-glutamine (Gibco, Thermo Fisher Scientific), 10 µM of sodium pyruvate (Sigma-Aldrich, Saint Louis, MO, USA) and of 1 mM glucose (Sigma-Aldrich, Saint Louis, MO, USA) at pH 7.4. The cells were then incubated 1 h at 37 °C in a CO_2_-free incubator for temperature and pH equilibration. The assay starts with the measurements of the basal oxygen consumption rate (OCR), the posterior addition of 1 μM oligomycin, 1.5 μM carbonyl cyanide-4-trifluoromethoxy phenylhydrazone (FCCP) and 0.5 μM rotenone/0.5 μM antimycin A were used to estimate the ATP production rate, the maximal mitochondrial respiratory capacity and the non-mitochondrial OCR, respectively. For the glycolytic assay, after 7 days of culture, the cells were washed twice with Seahorse XF DMEM phenol red-free (Agilent Technologies) containing 20 µM of L-glutamine (Gibco, Thermo Fisher Scientific), 10 µM of sodium pyruvate (Sigma-Aldrich, Saint Louis, MO, USA), 1 mM glucose (Sigma-Aldrich, Saint Louis, MO, USA) and 25 µM of HEPES (Gibco, Thermo Fisher Scientific). The cells were then incubated 1 h at 37 °C in a CO_2_-free incubator for temperature and pH equilibration. The assay started with the measurement of the basal glycolytic Proton Efflux Rate (glycoPER), followed by addition of 0.5 μM rotenone/0.5 μM antimycin A enabling calculation of mitochondrial-associated acidification and compensatory glycolysis. Eventually, the addition of 50 mM 2-deoxy-D-glucose was used to estimate the non-glycolytic PER. The first measurement of this assay is the basal glycolysis. Data were analyzed with Wave (v. 2.6; Agilent Technologies, Santa Clara, CA, USA) and the values were normalized with total protein values, measured by the Bradford method as described [11]. The cellular bioenergetics assays were performed at least 4 times in duplicates.

### 2.7. Statistical Analysis

Data are shown as mean ± SEM. One-way ANOVA with Dunnett’s multiple comparisons test or Kruskal–Wallis with Dunn’s multiple comparisons test were used to detect differences between groups. Statistically significant differences between these two groups are indicated by * *p* < 0.05 or ** *p* < 0.01. Statistical calculations were performed using the Graphpad Prism analysis software.

## 3. Results and Discussion

### 3.1. Upregulation of Hydrogen-Sulfide-Producing Enzymes Occurs during the Accumulation of ‘Driver’ Pathway Mutations

In the current work, we used organoids with various mutations (summarized in Table 1) to evaluate the effect of these mutations on the expression of various H_2_S-producing and H_2_S-metabolizing enzymes. In prior studies of various colon cancer cell lines and human colon cancer tissues, the expression of CBS and 3-MST was found [3,15]. In other cancers—e.g., ovarian cancer, lung cancer, breast cancer and prostate cancer—CBS, CSE and/or 3-MST upregulation was reported previously (reviewed in [3]).

**Figure 1:**
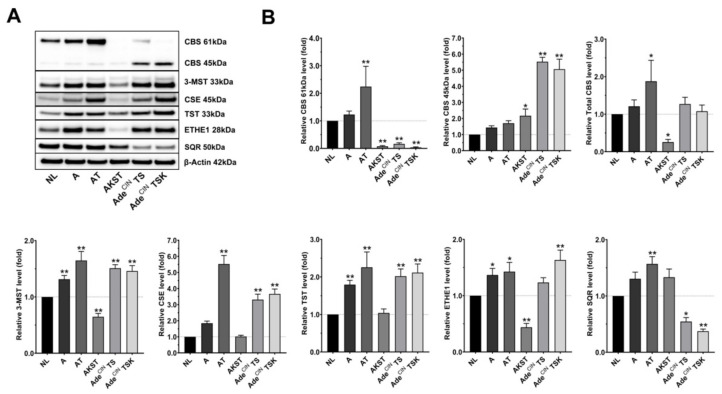
**Upregulation of H_2_S-producing and H_2_S-metabolizing enzymes occurs during the accumulation of ‘driver’ pathway mutations in human intestinal epithelial cell organoids.** Western blot analysis (**A**) and densitometric quantification (**B**) of H_2_S-producing (CBS, 3-MST, CSE) and H_2_S-metabolizing (TST, ETHE1, SQR) enzymes in NL, A, AT, AKST, Ade^CIN^ TS and Ade^CIN^ TSK organoids after 7 days in culture. Data represent mean ± SEM of at least 5 independent experiments. * *p* < 0.05, ** *p* < 0.01 compared to control.

Figure 1 and Table 1 show the changes in protein expression of the principal H_2_S-producing and H_2_S-metabolizing enzymes in the various organoid clones. Regarding the H_2_S-producing enzymes: CBS full-length (61 kDa) tends to show an increase in organoid A (i.e., after the loss of the APC gene) and is significantly elevated in AT organoids (loss of APC gene, and mutation of TP53). However, full-length CBS is significantly downregulated in AKST, Ade^CIN^ TS and Ade^CIN^ TSK organoids. Importantly, in the same organoids, a significant increase of the truncated form of CBS (45 kDa) can be seen (Figure 1). This truncated form of CBS represents a proteolytic cleavage product that lacks the C-terminal regulatory domain [16,17,18]. The 45 kDa CBS is constitutively (maximally) active, i.e., its activity is no longer dependent on the allosteric CBS activator S-adenosyl-methionine (SAM), which binds to the C-terminal region of the enzyme [16,17,18]. When we quantify the expression of total CBS (full length plus the truncated form) an upregulation can be seen in the AT organoids and a downregulation in the AKST organoids. However, even total CBS is not significantly upregulated in Ade^CIN^ TS and Ade^CIN^ TSK organoids., We should emphasize the fact that these organoids, for the most part, contain a more catalytically active form of the protein, which would be expected to yield higher rates of H_2_S production. Importantly, the appearance of the truncated form of CBS has previously been observed in human colon cancer cell lines (e.g., HCT116) [3,19] as well as in various pathophysiological conditions associated with CBS upregulation and H_2_S overproduction, for instance in the Down syndrome brain [20].

3-MST was significantly overexpressed in all cancer-related organoids except AKST; CSE expression was significantly upregulated in AT, Ade^CIN^ TS and Ade^CIN^ TSK organoids (Figure 1).

With respect to the expression of H_2_S-metabolizing enzymes: TST is significantly upregulated in A, AT, Ade^CIN^ TS and Ade^CIN^ TSK organoids; ETHE1 is markedly overexpressed in A, AT and Ade^CIN^ TSK and downregulated in AKST organoids; SQR is significantly upregulated in AT and downregulated in Ade^CIN^ TS and Ade^CIN^ TSK organoids (Figure 1).

Taken together, these results reveal the upregulation of various H_2_S-generating pathways (chiefly CBS and 3-MST), which begins at the earliest stage of colon cancer development (APC mutation). These findings are in line with prior observations in various colon cancer cell lines and in human colon cancer clinical specimens, demonstrating a marked upregulation of CBS, which starts already at the stage of APC mutations and adenoma formation [1,3,11,19,21,22,23,24,25,26]—as well as the (more modest and more variable) upregulation of 3-MST [11,12,15,23,24,27,28] in various experimental models of colon cancer. With respect to the regulation of the various H_2_S-metabolizing enzymes, the published body of data has been variable [29,30,31]; based on the findings of the current study, we hypothesize that the simultaneous upregulation of some of these enzymes in the more advanced organoids may serve as a potential compensatory mechanism to counter-regulate the increased H_2_S generation. With respect to TST/rhodanese, it should also be mentioned that—although it is generally viewed as one of the H_2_S-metabolizing enzymes—its biological roles are complex. In the context of the so-called thiosulfate cycle, thiosulfate is metabolized to produce H_2_S and sulfite, which, in turn, can be subsequently recycled into thiosulfate. In this cycle, 3-MST produces thiosulfate from sulfite and 3-mercaptopyruvate, and TST/rhodanese metabolizes thiosulfate into H_2_S and sulfite [32,33]. Thus, TST/rhodanese, in concert with 3-MST, may have both H_2_S-generating and H_2_S-degrading roles, depending on the biochemical context.

Importantly, the current data also reveal that at more advanced stages of the disease a conversion of CBS from the full length to the truncated form occurs, perhaps as a consequence of upregulation of trypsin and various other proteolytic enzymes [34,35,36,37] in the colon cancer cells.

### 3.2. H_2_S Production Is Increased in Ade^CIN^ TS and Ade^CIN^ TSK Organoids and Correlates with Increased Proliferation

**Figure 2:**
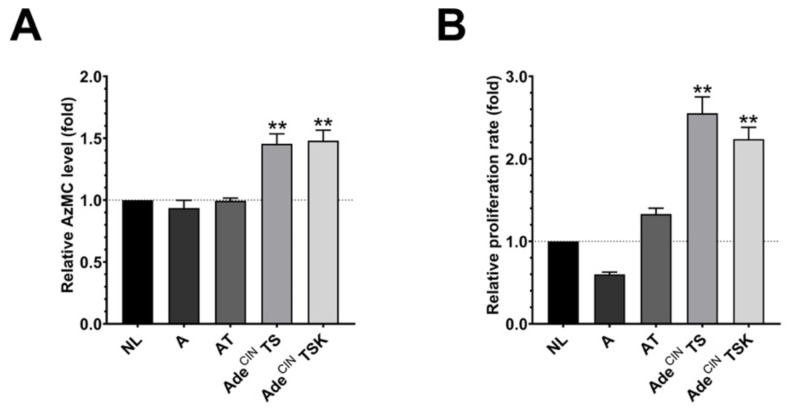
**Increased H_2_S production and increased cell proliferation in Ade^CIN^ TS and Ade^CIN^ TSK organoids.** (**A**) AzMC-aided live cell imaging quantification of NL, A, AT, Ade^CIN^ TS and Ade^CIN^ TSK organoids after 7 days in culture. (**B**) Cell proliferation rate of NL, A, AT, Ade^CIN^ TS and Ade^CIN^ TSK organoids after 7 days in culture. Data represent mean ± SEM of at least 4 independent experiments. ** *p* < 0.01 compared to control.

Since several H_2_S-producing enzymes are upregulated in the organoids, we next investigated the H_2_S production in A, AT, Ade^CIN^ TS and Ade^CIN^ TSK organoids by live cell imaging using the H_2_S-reactive dye AzMC. Although A, AT, Ade^CIN^ TS and Ade^CIN^ TSK organoids show an upregulation of various H_2_S-producing enzymes, this did not result in a corresponding increase in the H_2_S/AzMC signal in the A and AT organoids (Figure 2A), likely due to the fact that several H_2_S-metabolizing enzymes were also upregulated in the same organoids. However, significantly elevated H_2_S levels were detected in the most advanced organoids studied: Ade^CIN^ TS and Ade^CIN^ TSK (Figure 2A). Interestingly, in these two organoids, SQR expression is suppressed (as opposed to the increase in SQR expression seen in the organoids with earlier stage mutations) (Figure 1). Since SQR significantly contributes to cellular H_2_S utilization, a causative relationship between SQR downregulation and increased cellular H_2_S levels in Ade^CIN^ TS and Ade^CIN^ TSK organoids is possible but remains to be further investigated.

The high cellular H_2_S levels in Ade^CIN^ TS and Ade^CIN^ TSK were associated with higher proliferation rates (Figure 2B). Overall, a good correlation between H_2_S generation and organoid proliferation rate was noted across the organoids investigated—in line with the theory that increased H_2_S generation in colon cancer cells is a stimulator of cancer cell proliferation, via a variety of mechanisms—including cellular signaling and bioenergetics, as reviewed recently in [3].

### 3.3. Upregulation of the Wnt/β-Catenin Pathway Occurs during the Accumulation of ‘Driver’ Pathway Mutations

**Figure 3:**
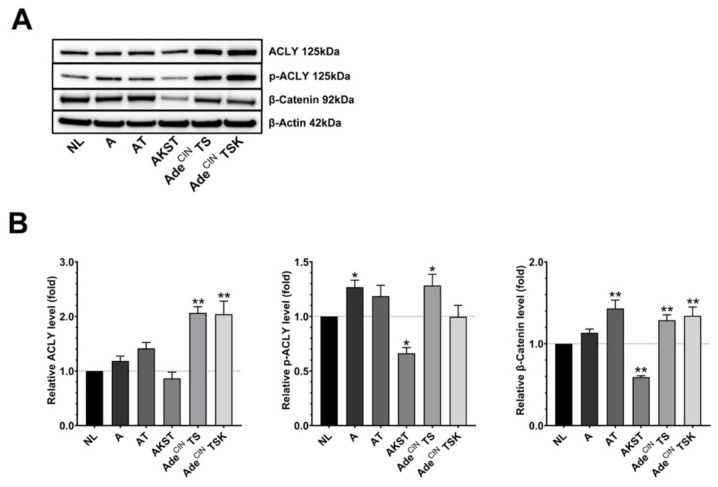
**Upregulation of the Wnt/β-catenin pathway occurs during the accumulation of ‘driver’ pathway mutations in human intestinal epithelial cell organoids.** (**A**,**B**) Western blot analysis of ACLY, p-ACLY and β-Catenin in NL, A, AT, AKST, Ade^CIN^ TS and Ade^CIN^ TSK organoids after 7 days in culture. Data represent mean ± SEM of at least 4 independent experiments. * *p* < 0.05, ** *p* < 0.01 compared to control.

We recently demonstrated that cancer cell-derived H_2_S, produced by CBS and 3-MST promotes EMT of human colon cancer cells via the stimulation of the Wnt/β-catenin pathway [11]. Accordingly, we investigated the expression of some key molecules of the Wnt/β-catenin pathway; ACLY, p-ACLY and β-catenin in the current organoid system. In general, all organoids (except AKST) overexpress one or more of these molecules, with higher upregulation seen in the more advanced organoids (Figure 3). Concerning A organoids, we showed a significant increase in p-ACLY expression and a trend for increased expression of ACLY and β-catenin. AT organoids exhibited significant overexpression of β-catenin and a trend for increased ACLY and p-ACLY. ACLY, p-ACLY and β-catenin were all significantly overexpressed in Ade^CIN^ TS organoids. Finally, Ade^CIN^ TSK organoids had a significant increase of ACLY and β-catenin. These results confirm previous findings that Wnt/β-catenin pathway activation occurs during colorectal cancer development [38,39]; based on prior studies in human colon cancer cell lines [11,21], upregulation of H_2_S generation may be a potential contributing factor to this process, although the effect of H_2_S biosynthesis inhibitors has not been assessed in the current set of experiments. Interestingly, prior studies have implicated the role of the Wnt/β-catenin pathway in the upregulation of CSE in several human colon cancer cell lines [40]; whether this mechanism is operative in the current system remains to be further investigated, but there appears to be a correlation: both CSE upregulation and the induction of various Wnt/β-catenin pathway components were found to be most pronounced in the same organoids: AT, Ade^CIN^ TS and Ade^CIN^ TSK (Figure 1 and Figure 3).

### 3.4. Upregulation of Cellular Bioenergetics and Glycolysis in Ade^CIN^ TS and Ade^CIN^ TSK Organoids

**Figure 4:**
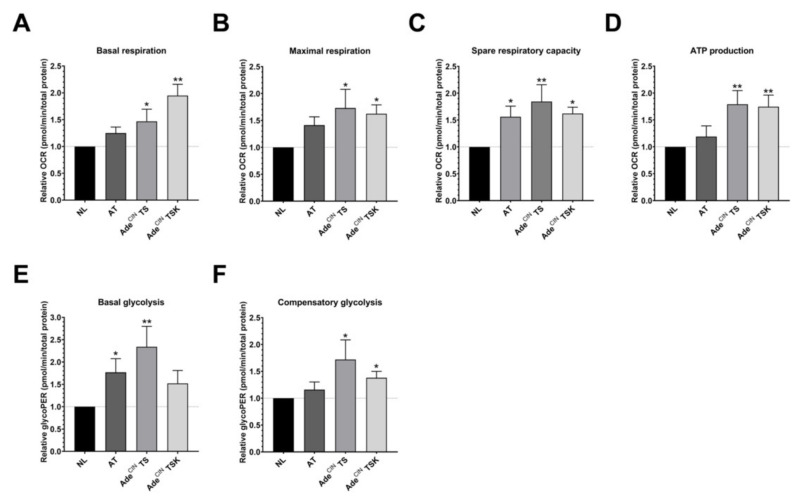
**Upregulation of cellular bioenergetics in Ade^CIN^ TS and Ade^CIN^ TSK organoids.** Mitochondrial respiration and glycolysis of NL, AT, Ade^CIN^ TS and Ade^CIN^ TSK organoids after 7 days in culture, assessed by Extracellular Flux Analysis. (**A**) Basal respiration. (**B**) Maximal respiration. (**C**) Spare respiratory capacity. (**D**) ATP production. (**E**) Basal glycolysis. (**F**) Compensatory glycolysis. Data represent mean ± SEM of at least 4 independent experiments. * *p* < 0.05, ** *p* < 0.01 compared to control.

Results presented in the previous section demonstrated that Ade^CIN^ TS and Ade^CIN^ TSK organoids exhibit increased levels of H_2_S. Since H_2_S overproduction in cancer cells is known to stimulate mitochondrial respiration and cellular bioenergetics [3,41], we next measured cellular bioenergetics in the current organoid system. We compared control (normal epithelial cell) organoids with a selected organoid where H_2_S levels were not yet increased (AT) and with the two organoids where significantly elevated H_2_S levels were detected (Ade^CIN^ TS and Ade^CIN^ TSK). Basal respiration, maximal respiration, spare respiratory capacity and ATP production were all found to be upregulated in the Ade^CIN^ TS and Ade^CIN^ TSK organoids (Figure 4). Moreover, basal glycolysis and compensatory glycolysis were both upregulated in Ade^CIN^ TS organoids, contrary to AT and Ade^CIN^ TSK organoids in which only basal glycolysis or compensatory glycolysis are upregulated, respectively. We hypothesize that this difference may explain the slightly higher proliferation rate of Ade^CIN^ TS organoids compared to Ade^CIN^ TSK organoids (Figure 2B).

The ‘classical’ view of the Warburg effect (i.e., that cancer cells tend to switch their metabolism from oxidative phosphorylation to glycolysis) has been revised over recent years. According to the current concept, cancer cells have a tendency to upregulate all available pathways of metabolism, when the pathways are functionally operative, i.e., there can be an upregulation of oxidative phosphorylation (when the mitochondria are intact and O_2_ is available), as well as an upregulation of glycolysis in the same cancer cell [42,43,44,45]. The current findings are in agreement with this concept, but also demonstrate that the relative upregulation of glycolysis is not a uniform effect and may be linked to specific driver mutations: the comparison of the Ade^CIN^ TS and Ade^CIN^ TSK organoids (the latter having an additional mutation, a knock-in mutation KRASG12V) suggests that the upregulation of glycolysis may be the highest when TP53 mutation and loss of SMAD are present, but when an additional KRAS mutation is also introduced, then the net upregulation of glycolysis becomes less pronounced. Prior studies have already linked TP53 and KRAS mutations to the activation of glycolysis in colon cancer cells [46,47], but the functional interaction between remains to be explored.

### 3.5. Pharmacological Inhibition of H_2_S Biosynthesis in Ade^CIN^ TS and Ade^CIN^ TSK Suppresses Cell Proliferation

**Figure 5:**
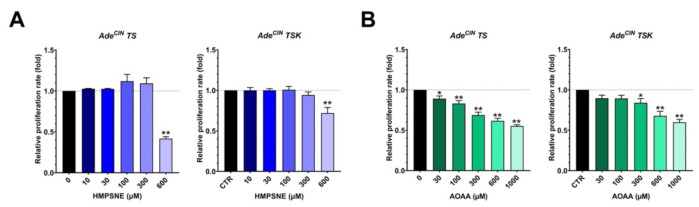
**Pharmacological inhibition of H_2_S biosynthesis in Ade^CIN^ TS and Ade^CIN^ TSK attenuates cell proliferation.** (**A**) Relative proliferation rate of Ade^CIN^ TS and Ade^CIN^ TSK in the presence of increasing concentrations of HMPSNE for 48 h. (**B**) Relative proliferation rate of Ade^CIN^ TS and Ade^CIN^ TSK in the presence of increasing concentrations of AOAA for 48 h. Data represent mean ± SEM of at least 4 independent experiments. * *p* < 0.05, ** *p* < 0.01 compared to control.

Next, we investigated if the pharmacological inhibition of H_2_S biosynthesis in Ade^CIN^ TS and Ade^CIN^ TSK organoids can affect cell proliferation. HMSPNE and AOAA were used to inhibit 3-MST and CBS/CSE, respectively. Figure 5 shows that both inhibitors produce a significant, concentration-dependent decrease in the proliferation rate of both organoids. These results are in line with prior observations in various human colon cancer cell lines (e.g., HCT116, CaCo2, HT-29) demonstrating inhibitory effects of CBS and/or 3-MST inhibition and/or silencing on cell proliferation rate—in agreement with the stimulatory effect of endogenously produced H_2_S on cancer cell proliferation [1,2,3,21].

However, as discussed previously [3,41,48,49,50,51], the effects of H_2_S are bell-shaped; elevation of H_2_S levels above and beyond a concentration optimum starts to exert adverse effects and can suppress cell proliferation. This bell-shaped concentration-response is the likely explanation for why other reports find that (relatively high concentrations of) pharmacological H_2_S donors can decrease colon cancer cell proliferation and can also induce cytotoxic or cytostatic effects.

### 3.6. Effect of 3-MST Inhibition on the Expression of Various H_2_S-Producing and H_2_S-Metabolizing Enzymes and on the Wnt/β-Catenin Pathway in Ade^CIN^ TS and Ade^CIN^ TSK Organoids

**Figure 6:**
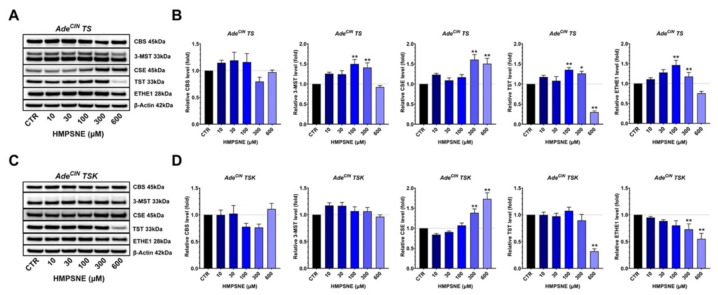
**Effect of pharmacological inhibition of 3-MST on the expression of various H_2_S-producing and H_2_S-metabolizing enzymes in Ade^CIN^ TS and Ade^CIN^ TSK organoids**. (**A**,**B**) Western blot analysis of H_2_S-producing and H_2_S-metabolizing enzymes in Ade^CIN^ TS organoids in presence of increasing concentrations of HMPSNE. (**C**,**D**) Western blot analysis of H_2_S-producing and H_2_S-metabolizing enzymes in Ade^CIN^ TSK organoids in presence of increasing concentrations of HMPSNE. Analysis was performed at 48 h. CBS refers to the 45 kDa, truncated form, which was the CBS isoform predominantly present in these organoids. Data represent mean ± SEM of at least 4 independent experiments. * *p* < 0.05, ** *p* < 0.01 compared to control.

In the next set of experiments, we tested the effect of the 3-MST inhibitor HMPSNE on the expression of various H_2_S-producing and -metabolizing enzymes and of some key regulators of the Wnt/β-catenin pathway in Ade^CIN^ TS and Ade^CIN^ TSK organoids. In Ade^CIN^ TS organoids, 3-MST inhibition increased the expression of 3-MST itself (possibly suggesting a self-amplifying positive feedforward cycle between H_2_S generation and 3-MST expression), but also induced a significant overexpression of CSE, TST and ETHE1 (Figure 6A,B). The increase in the expression of 3-MST and CSE, induced by lower concentrations of HMPSNE, may explain the slight tendency for increased Ade^CIN^ TS proliferation observed in the presence of lower concentrations (100 and 300 µM) of HMPSNE (Figure 5A). A downregulation of TST was observed at 600 µM HMPSNE. No significant effects of the 3-MST inhibitor were detected on CBS expression.

In Ade^CIN^ TSK organoids, we observed a CSE upregulation in the presence of 300 and 600 µM of HMPSNE, a significant decrease in TST and ETHE1 expression with the higher concentrations of HMPSNE, and there was no significant effect on CBS or 3-MST expression (Figure 6C,D). CSE overexpression and the TST and ETHE1 downregulation in presence of 600 µM HMPSNE in Ade^CIN^ TSK organoids may explain the relatively less pronounced inhibitory effect of HMPSNE on the proliferation rate of Ade^CIN^ TSK organoids compared to Ade^CIN^ TS (Figure 5A).

**Figure 7:**
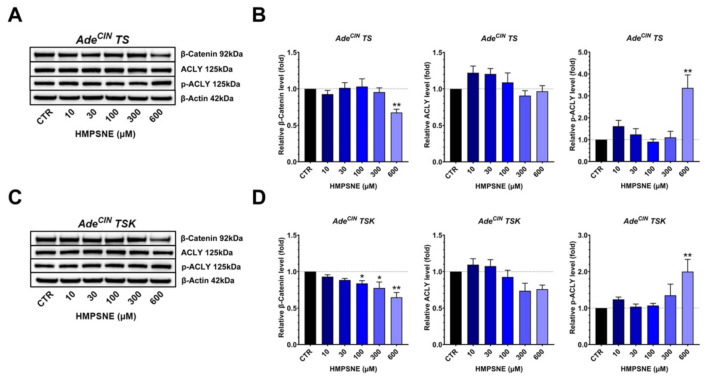
**Effect of pharmacological inhibition of 3-MST on the induction of the Wnt/β-catenin pathway in Ade^CIN^ TS and Ade^CIN^ TSK organoids**. (**A**,**B**) Western blot analysis of β-catenin, ACLY and p-ACLY in Ade^CIN^ TS organoids in the presence of increasing concentrations of HMPSNE for 48 h. (**C**,**D**) Western blot analysis of β-catenin, ACLY and p-ACLY in Ade^CIN^ TSK organoids in the presence of increasing concentrations of HMPSNE for 48 h. Data represent mean ± SEM of at least 4 independent experiments. * *p* < 0.05, ** *p* < 0.01 compared to control.

In both Ade^CIN^ TS and Ade^CIN^ TSK organoids, we found a significant suppression of β-catenin expression after treatment with 600 µM HMSPNE; however, a significant increase of p-ACLY was also noted at the same concentration of this inhibitor (Figure 7). These data confirm prior findings [11], suggesting that 3-MST upregulates the activation of the Wnt/β-catenin pathway, but indicate that pathways other than ACLY may be involved in this process in the current organoid system.

### 3.7. Effect of CBS Inhibition on the Expression of Various H_2_S-Producing and H_2_S-Metabolizing Enzymes and on the Wnt/β-catenin Pathway in Ade^CIN^ TS and Ade^CIN^ TSK Organoids

**Figure 8:**
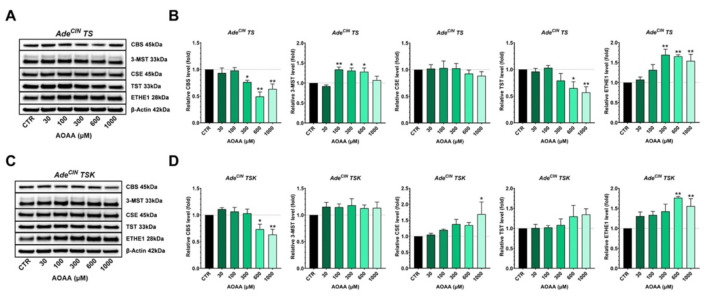
**Effect of pharmacological inhibition of CBS/CSE on the expression of various H_2_S-producing and -metabolizing enzymes in Ade^CIN^ TS and Ade^CIN^ TSK organoids**. (**A**,**B**) Western blot analysis of H_2_S-producing and H_2_S-metabolizing enzymes in Ade^CIN^ TS in the presence of increasing concentrations of AOAA for 48 h. (**C**,**D**) Western blot analysis of H_2_S-producing and -metabolizing enzymes in Ade^CIN^ TSK in the presence of increasing concentrations of AOAA for 48 h. Data represent mean ± SEM of at least 4 independent experiments. CBS refers to the 45 kDa, truncated form, which was the CBS isoform predominantly present in these organoids. * *p* < 0.05, ** *p* < 0.01 compared to control.

In the last set of experiments, Ade^CIN^ TS and Ade^CIN^ TSK organoids were incubated with increasing concentrations of the CBS/CSE inhibitor [9] AOAA and the expression of various H_2_S-producing and H_2_S-metabolizing enzymes, and some key regulators of the Wnt/β-catenin pathway were measured (Figure 8).

In Ade^CIN^ TS organoids, the expression of CBS and TST was significantly suppressed and ETHE1 expression was significantly increased in response to the highest concentrations of AOAA used. 3-MST expression was increased in the presence of 100, 300 and 600 µM of AOAA, while no significant effect of AOAA was observed on CSE expression (Figure 8A,B).

In Ade^CIN^ TSK organoids, a significant reduction on CBS expression and an upregulation of ETHE1 was noted at the highest AOAA concentrations. Additionally, an overexpression of CSE was observed at the highest concentration of the inhibitor used, while no significant impact of AOAA on 3-MST or on TST expression was detected (Figure 8C,D). These data suggest that CBS-derived H_2_S plays a variable effect on the expression of various enzymes involved in H_2_S generation and metabolism; compared to the role of 3-MST-derived H_2_S, the effects were less pronounced and less consistent.

**Figure 9:**
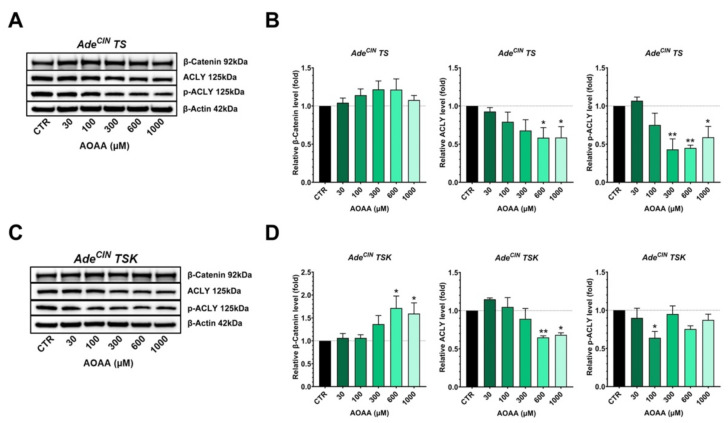
**Effect of pharmacological inhibition of CBS/CSE on the induction of the Wnt/β-catenin pathway in Ade^CIN^ TS and Ade^CIN^ TSK organoids**. (**A**,**B**) Western blot analysis of β-catenin, ACLY and p-ACLY in Ade^CIN^ TS in the presence of increasing concentrations of AOAA for 48 h. (**C**,**D**) Western blot analysis of β-Catenin, ACLY, and p-ACLY in Ade^CIN^ TSK in the presence of increasing concentrations of AOAA for 48 h. Data represent mean ± SEM of at least 4 independent experiments. * *p* < 0.05, ** *p* < 0.01 compared to control.

In Ade^CIN^ TS organoids, we observed a significant decrease on ACLY and p-ACLY expression in the presence of the highest concentrations of AOAA, while no significant effect was observed on β-catenin expression (Figure 9). Similarly, a downregulation of ACLY was also identified in Ade^CIN^ TSK organoids in the presence of the highest concentration of AOAA and a significant decrease on p-ACLY expression was observed at 100 µM AOAA. However, in Ade^CIN^ TSK organoids an upregulation of β-catenin expression was seen with 600 µM AOAA. Taken together, the effect of CBS/CSE inhibition in Ade^CIN^ TS and Ade^CIN^ TSK is inconsistent on the Wnt/β-catenin pathway.

It should be pointed out that CBS and CSE are major sources of ‘free H_2_S’, while 3-MST predominantly generates reactive polysulfides rather than H_2_S (although in a cellular environment both species are likely to be present) [51]. Moreover, the subcellular localization of these enzymes is different, with CSE mainly cytosolic, CBS cytosolic, but also mitochondrially translocated in cancer cells, and 3-MST equally distributed to mitochondria and cytosol [51]. Thus, the biological role of CBS, CSE and 3-MST may well be different, depending on their localization and the mixture of reactive sulfur species produced by them. In addition, in the current experimental system, all three H_2_S-producing enzymes contribute to the overproduction of H_2_S (as well as other reactive sulfur species, which were not measured here), and we have not assessed the simultaneous inhibition of CBS/CSE/3-MST on various functional parameters or signaling pathways—but it is likely that such an approach would produce a more pronounced suppression of proliferation and perhaps a combined inhibitory effect on ACLY activation as well as the activation of the Wnt/β-catenin signaling pathway. This combined approach remains to be tested, either using pharmacological or genetic tools. As with all pharmacological inhibitors, with the inhibitors used in the current study (AOAA and HMPSNE), potential issues of non-specific pharmacological effects should also be considered. For instance, AOAA is known to inhibit certain transaminases, some of which play a role in colon cancer pathobiology. Nevertheless, the approach used here can be considered the most feasible one according to the current state-of-the-art, given the well-known limitations [3,15,18,25,28,48,49,50,51,52] on the availability of potent, selective and cell-permeable inhibitors of the various H_2_S-producing enzymes.

Several questions remain to be addressed with respect to the mechanism of the upregulation of the various H_2_S-pathway enzymes during intestinal carcinogenesis. One of these questions is the following: Is the induction of these enzymes a cause or a consequence of the increased cell proliferation rate during the sequential accumulation of the various driver mutations? It has been known for two decades that cell proliferation and CBS expression are coordinately regulated; Jan Kraus and colleagues observed that proliferating cells have higher CBS expression than non-proliferating ones and proposed a redox-based mechanism for the upregulation of CBS, perhaps via Sp1 [53,54]. However, the exact mechanisms of this process have not been defined. The upregulation of the H_2_S pathway enzymes in the current experimental model, in response to the introduction of the various driver mutations, may occur either directly (via the regulation of various signaling pathways that regulate the cellular levels of these enzymes) or indirectly (via increasing cell proliferation rate, which, in turn, may upregulate the cellular levels of these enzymes). Scenarios involving positive feedforward cycles are also possible. For instance, driver mutations and/or proliferation itself may upregulate H_2_S-generating enzymes, and the H_2_S produced by them may, in turn, further stimulate cellular bioenergetics and proliferation. Indeed, it is well known that administration of low concentrations of pharmacological H_2_S donors to colon cancer cells can increase cellular bioenergetics and stimulate cell proliferation, even in the absence of any additional mutations or stimulation of signaling pathways [3,23,41,52].

A second, related question relates to the mode of regulation of the various H_2_S pathway enzymes (i.e., the relative importance of transcriptional vs. post-transcriptional mechanisms). A previous study [8] conducted RNAseq analysis of colorectal adenoma and primary colorectal cancer tissues, as well as two of the organoids (‘A’ and ‘AKST’) used in the current project. The mRNA expression data are shown in Table 2. These data do not demonstrate marked differences in the mRNA levels, indicating that the changes in protein levels of the H_2_S-producing enzymes detected in the current model system must be primarily due to post-transcriptional (rather than transcriptional) mechanisms. Indeed, CBS is known to be a subject of a significant post-transcriptional regulation at the level of protein stability via ubiquitination and proteasomal degradation [55,56].

It should be also emphasized that the expression of H_2_S-producing enzymes may not always directly correlate with cellular H_2_S generation. For instance, the intracellular (re)distribution of these enzymes (e.g., mitochondrial translocation of CBS and/or CBS oligomerization/polymerization [18,51]) may also affect their cellular regulatory roles. Potential post-translational modifications of these enzymes (e.g., the glutathionylation of CBS) may also affect its function. Changes in the cellular levels of the allosteric activator of CBS, S-adenosyl-methionine (SAM) during intestinal carcinogenesis may also be important. In fact, in a prior study, intracellular SAM levels were found to be elevated in the polyps of the ApcMin/+ mice [57]. Moreover, in the intestinal polyps of patients an increase in methionine adenosyltransferase 2A mRNA levels was reported [57]. Elevation in SAM levels may increase the catalytic activity of full-length CBS in the adenomas or early stages of colorectal cancer. However, in later stages of colorectal cancer, our data show that the predominant form of CBS is the truncated form. This form no longer has the C-terminal regulatory domain, and therefore it is in a maximally activated stage and is no longer dependent on the activating effect of SAM. In a previous study utilizing HCT116 cells (a human colon cancer cell line, which contains a mixture of the full-length and cleaved CBS) we tested the effect of exogenous SAM addition, and SAM induced an increase in H_2_S generation in these cells and exerted functional effects (stimulation of cellular bioenergetics and proliferation) that are consistent with the elevation of H_2_S [19]. The intracellular levels of SAM in the current organoid system remain to be investigated. Likewise, enzymes and processes that regulate substate availability—e.g., the expression and activity of membrane transporters for cyst(e)ine [58,59], or cysteine aminotransferase (CAT), the enzyme that produces 3-MP, the substrate of 3-MST [60]—remain to be investigated in future studies.

## 4. Conclusions

Taken together, the current study demonstrates that consecutive ‘driver’ mutations produce gradual upregulations in CBS expression, in particular its truncated (45 kDa) form and in 3-MST as well as CSE expression. In organoids harboring early mutations of the Vogelstein sequence, the upregulation of these H_2_S-generating enzymes occurs together with the upregulation of various H_2_S-metabolizing enzymes, and live cell imaging does not detect significant elevations in cellular H_2_S levels. However, in organoids harboring later-stage mutations, expression of the H_2_S-producing enzymes further increases, while the expression of certain H_2_S-metabolizing enzymes (e.g., SQR) decreases, and a detectable increase in cellular H_2_S levels can be demonstrated. These late-stage organoids also exhibit significantly increased proliferation rate and upregulation of cellular bioenergetics (oxidative phosphorylation, and in some cases glycolysis as well). Previous studies have demonstrated that these later-stage organoids assume aggressive phenotypes, in line with the classic adenoma-carcinoma sequence model. Ade^CIN^ TS-organoids display highly polarized morphology, but fail to macro-metastasize to the liver after injection into the spleen [8]. However, after the KRASG12V mutation is additionally introduced (Ade^CIN^ TSK organoids) the organoids assume cribriform-like dysplastic structures in vitro and are capable of forming large metastatic tumors displaying histologic malignancy in vivo [8].

Although the molecular mechanism of the metastatic progression remains unknown, these results suggest that accumulating driver pathway mutations are essential for colon carcinogenesis and upregulation of the H_2_S pathway, and the increase in cellular H_2_S generation in the two most aggressive organoids studied may contribute to the high degree of aggressiveness and invasiveness of these colon cancer cells. Moreover, upregulation of ACLY and the Wnt/β-catenin pathway indicates that at the advanced stage of cancer, cells in the organoids undergo EMT, an essential element of invasiveness and metastasis. Pharmacological inhibition of CBS/CSE or 3-MST attenuates the proliferation of the organoids and modulates the expression of various H_2_S-producing and H_2_S-metabolizing enzymes and also modulates some (but not all) elements and regulators of EMT.

Taken together, the current data further strengthen the view that increased H_2_S generation, by promoting cancer cell bioenergetics, signaling and cellular dedifferentiation, plays a significant role in the pathogenesis of colon cancer and supports the view that pharmacological inhibition of this process may be of potential future therapeutic value.

## Figures and Tables

**Table 1:** Clones, associated mutations, and resultant changes in the expression (protein levels, determined by Western blotting) of H_2_S-producing enzymes.

Clones	Mutation(s) & Chromosomal Instability	Cancer Stage	CBS 45 kDa ^1^	CBS 61 kDa ^1^	3-MST ^1^	CSE ^1^
**NL**	Normal control	Healthy	100	100	100	100
**A**	Loss of APC gene	Early adenoma	143 ± 11	123 ± 12	131 ± 7	185 ± 13
**AT**	Loss of APC gene Indel mutation of TP53, overexpression of a truncated stabilized form of TP53	Carcinoma	170 ± 16	224 ± 74	165 ± 16	552 ± 53
**AKST**	Loss of APC gene Knock-in mutation KRAS^G12V^ Indel mutation and loss of SMAD4 protein Indel mutation of TP53, overexpression of a truncated stabilized form of TP53	Carcinoma	217 ± 42	8 ± 3	65 ± 6	102 ± 7
**Ade^CIN^ TS**	Indel mutation of TP53, overexpression of a truncated stabilized form of TP53 Indel mutation and loss of SMAD4 protein Chromosomal instability	Carcinoma (non-metastatic)	553 ± 27	16 ± 4	151 ± 6	331 ± 34
**Ade^CIN^ TSK**	Indel mutation of TP53, overexpression of a truncated stabilized form of TP53 Indel mutation and loss of SMAD4 protein Knock-in mutation KRAS^G12V^ Chromosomal instability	Carcinoma (metastatic)	506 ± 62	4 ± 2	146 ± 10	367 ± 30

^1^ protein expression (mean ± SEM), quantified by Western blotting, expressed as % of normal control.

**Table 2:** Changes in the expression of H_2_S-producing and H_2_S-metabolizing enzymes during colorectal carcinogenesis (mRNA expression data) ^1^.

	Adenoma (*n* = 5)	Primary Colorectal Cancer (*n* = 7)	Metastatic Colorectal Cancer (*n* = 2)	Organoid ‘A’ (*n* = 1)	Organoid ‘AKST’ (*n* = 2)
**CBS**	88 ± 24	118 ± 27	90 ± 10	112	69 ± 8
**3-MST**	99 ± 7	95 ± 5	108 ± 8	144	100 ± 1
**CSE**	101 ± 21	96 ± 11	112 ± 25	95	80 ± 14
**ETHE1**	113 ± 30	88 ± 18	59 ± 31	171	85 ± 2
**TST**	105 ± 7	81 ± 9	102 ± 27	142	89 ± 15

^1^ mRNA levels (mean ± SEM), quantified by the Affymetrix Human Gene Expression Array, expressed as % of normal control. Data reported previously by Matano and colleagues [8] and retrieved from the Gene Expression Omnibus Database (Accession # GSE57965).

## Data Availability

Raw data generated in the current project are available, upon request, from the author of correspondence (C.S.).
